# Inflammation and nutritional status in relation to mortality risk from cardio-cerebrovascular events: evidence from NHANES

**DOI:** 10.3389/fnut.2024.1504946

**Published:** 2024-12-12

**Authors:** Chengzhi Hou, Xuanchun Huang, Jie Wang, Cong Chen, Chao Liu, Shuyuan Liu, Hongping Li

**Affiliations:** ^1^Guang’anmen Hospital, China Academy of Chinese Medicine Sciences, Beijing, China; ^2^College of Traditional Chinese Medicine, Hubei University of Chinese Medicine, Wuhan, China

**Keywords:** inflammation, nutrition, cardio-cerebrovascular events, cardiovascular mortality, all-cause mortality, NHANES

## Abstract

**Objective:**

Inflammation and nutritional status are closely associated with the mortality risk of survivors of cardio-cerebrovascular events. This study aims to evaluate the relationship between inflammation and nutritional indices and mortality among, identifying the most predictive indices.

**Methods:**

This study included cohort data of the survivors of major adverse cardiovascular and cerebrovascular events (MACCE) from the National Health and Nutrition Examination Survey (NHANES) in 1999–2010. MACCE is defined as a composite of myocardial infarction, heart failure and stroke, and at least one of the three events occurs. The main outcomes were all-cause mortality and cardiovascular mortality. Kaplan–Meier analysis and receiver operating characteristic curves were used to compare the correlation between seven inflammatory nutritional indices (such as Advanced Lung Cancer Inflammation Index, ALI) and mortality among the survivors. A multivariable-adjusted Cox regression and restricted cubic splines analysis identified the most predictive index, with the optimal number of nodes determined by the Akaike information criterion. Subgroup and sensitivity analyses were conducted to assess model stability.

**Results:**

A total of 2,045 MACCE survivors were included. The higher levels of ALI and serum albumin were significantly associated with lower risks of all-cause and cardiovascular mortality among these individuals. Increases in C-reactive protein to Lymphocyte Ratio, Neutrophil to Serum Albumin Ratio, Neutrophil-to-Lymphocyte Ratio, Systemic Immune-Inflammation Index (SII), and C-reactive protein were similarly correlated with higher mortality risk. ALI outperformed other indices, displaying a distinct L-shaped nonlinear relationship with both all-cause and cardiovascular mortality among MACCE survivors, with an inflection point at 90 indicating the lowest risk. To the left of this inflection, each unit increase in ALI was associated with a 1.3% decrease in all-cause and cardiovascular mortality risk among MACCE patients. To the right, the risk might increase by 0.2%, although the change was not statistically significant. Subgroup analyses and sensitivity analyses showed that the association between ALI and risk of mortality remained stable in most MACCE survivor populations.

**Conclusion:**

Routine and dynamic monitoring of ALI is helpful for clinicians to assess the mortality risk among MACCE survivors. Anti-inflammatory therapies and appropriate nutritional support are crucial for reducing mortality in these individuals.

## 1 Introduction

Cardio-cerebrovascular diseases are among the principal causes of mortality worldwide (1). Despite a 34.9% reduction in the age-standardized mortality rate from cardiovascular diseases globally over the past 30 years, the absolute number of deaths has increased by 7.4 million, reaching 19.8 million in 2022 (2). Currently, ischemic heart disease and stroke are the primary threats to the health and longevity of the middle-aged and elderly populations (3), with patients in the terminal stages of various cardiovascular diseases exhibiting a five-year survival rate of only 50% (4). The related complications, such as cognitive and motor disorders, seriously affect patients’ quality of life (5).

Inflammation and nutritional status are closely associated with the risk of death from cardio-cerebrovascular diseases (6). Immune-inflammatory responses are a critical pathological mechanism in these diseases. Following a cardiovascular event, the interaction between circulating platelets and neutrophils initiates the immune-inflammatory response, directly causing endothelial dysfunction (7). Moreover, the local infiltration of monocytes, lymphocytes, and macrophages promotes the secretion of pro-inflammatory factors, triggering a cascade of inflammatory responses that exacerbate arteriosclerosis in the cardio-cerebrovascular system. Chronic inflammation can also induce immunosuppression, exacerbating cardiac remodeling and adversely affecting outcomes (8–10). Notably, inflammatory responses disrupt energy metabolism, as evidenced by the rapid consumption of oxygen and nutrients in myocardial infarction sites, presenting challenges for leukocyte-mediated tissue repair (11). Additionally, the long-term burden of disease can lead to nutritional imbalances in survivors of cardio-cerebrovascular events, characterized by excessive consumption and redistribution of proteins and other nutrients (12–14).

Some circulating immune inflammatory cells, serum albumin, body mass index (BMI) and other indicators reflecting the inflammation and nutritional status of the body are closely related to the prognosis of cardio-cerebrovascular diseases. Advanced Lung Cancer Inflammation Index (ALI) is a new index, which incorporates serum albumin (ALB), BMI and the neutrophil-to-lymphocyte ratio (NLR), are closely linked to the prognosis of cardio-cerebrovascular diseases and was initially used to assess inflammation, nutritional status and prognosis of lung cancer patients (15, 16). Recent studies have shown its relevance to the prognosis of acute coronary syndrome, heart failure, hypertension, and stroke (17–20). Furthermore, a cross-sectional cohort study involving 12,615 participants identified the C-reactive protein to Lymphocyte Ratio (CLR) as a potential new inflammatory marker for myocardial infarction (21). Additionally, the Neutrophil to Serum Albumin Ratio (NPAR), NLR, and ALB have been reported to correlate with mortality in heart failure patients (22–24). Some composite inflammatory indices, such as the Systemic Immune-Inflammation Index (SII), which integrate platelet and neutrophil counts along with other inflammatory cell parameters, as well as single indices, C-reactive protein (CRP), have been associated with the occurrence of stroke and adverse outcomes such as vascular recurrence (25–27).

The above research suggests that nutrition and inflammation are closely related to the occurrence of MACCE. However, research on the relationship between nutritional inflammatory state and the risk of cardiovascular mortality remains limited, particularly in MACCE survivors. Additionally, the predictive accuracy of certain composite nutritional inflammatory indices, such as the Advanced Lung Cancer Inflammation Index (ALI), compared to single indicators, C-reactive protein (CRP), remains unclear. Therefore, building on previous related studies, we conducted an analysis using data from the National Health and Nutrition Examination Survey (NHANES). This study included composite indices such as ALI, CLR, NPAR, NLR, and SII, along with single inflammation and nutritional markers CRP and ALB. The aim was to evaluate the effects of different nutritional and inflammatory states on all-cause mortality and cardiovascular mortality in MACCE survivors, and to determine the most valuable and predictive indicators. It could offer new insights for managing and treating these patients, potentially enhancing their overall health management strategies and reducing mortality risks.

## 2 Methods

The study utilized the NHANES database conducted by the National Center for Health Statistics (NCHS). NHANES is a comprehensive survey designed to gather nationally representative data on the health and nutritional status of the civilian U.S. population, encompassing demographic, socioeconomic, dietary, and health-related information. To ensure the diversity of its sample, NHANES employs a stratified, multistage probability sampling method to select participants from across the nation. The study protocol was approved by the NCHS Research Ethics Review Board, and all participants provided written informed consent. Detailed information on the survey methodology can be accessed on the NHANES website.

### 2.1 Data and sample sources

**Figure 1:**
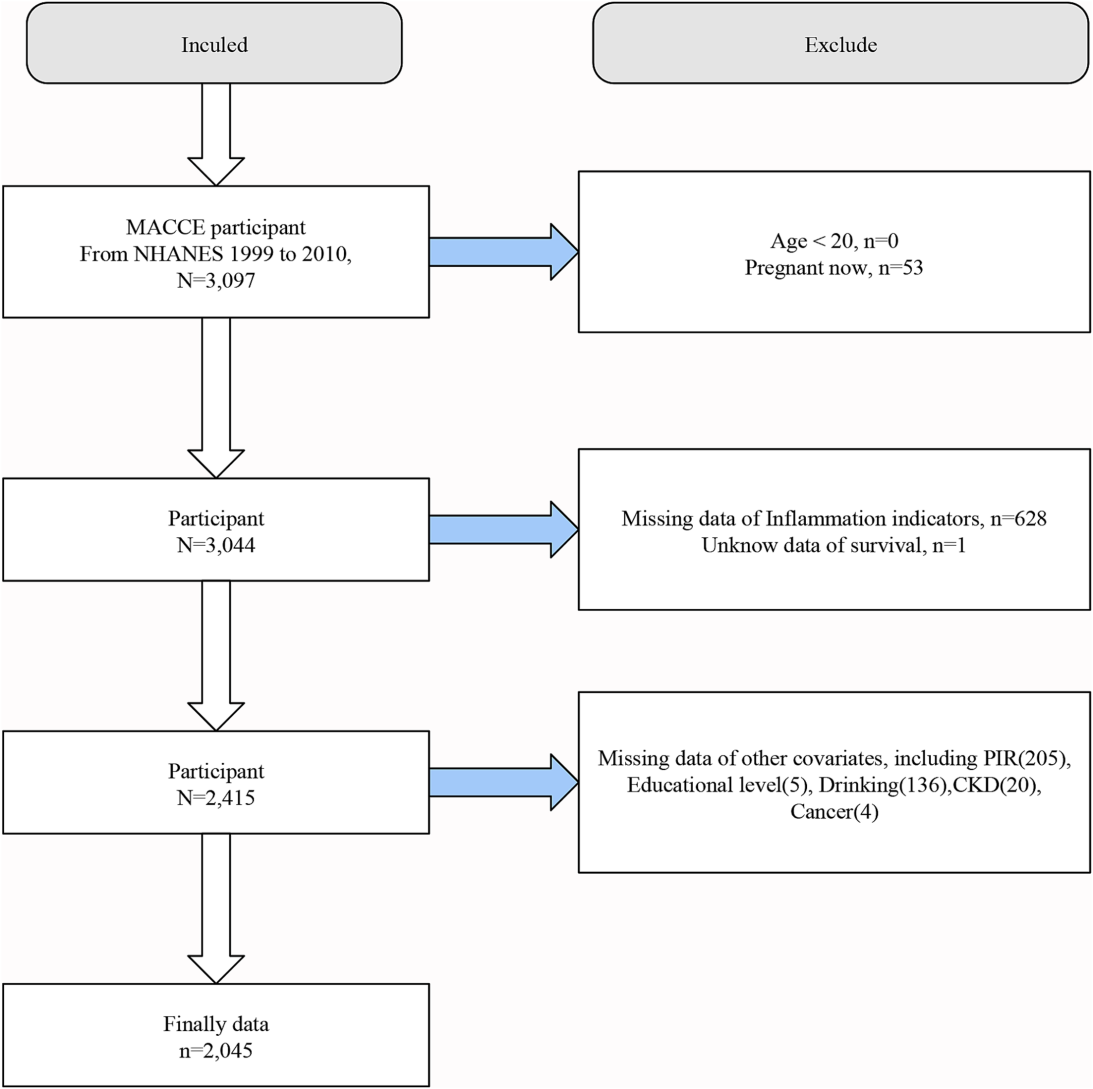
Flow chart of the participants selection process.

This study utilized publicly available data from NHANES spanning the years 1999–2010. The inclusion criteria for the study population were individuals who had survived major adverse cardiovascular and cerebrovascular events (MACCE), defined as a composite of myocardial infarction, heart failure and stroke, and at least one of the three events occurs (28). Screening criteria were as follows: (a) a clinical diagnosis of myocardial infarction; (b) a clinical diagnosis of heart failure; (c) a clinical diagnosis of stroke. Considering the reality of the NHANES database and requirements such as medical ethics, the following populations were excluded from this study. (a) individuals younger than 20 years old; (b) pregnant participants; (c) participants lacking data necessary to calculate Inflammation and nutritional indices; (d) unknow data of survival.; (e) participants missing any required covariates. In the NHANES database, the starting point of the adult group is often 20 years old, and in many regions and states of the United States, smoking and drinking are allowed only after 20 years old. After applying these criteria, a total of 2,045 individuals were included in the final analysis. The detailed screening process is illustrated in Figure 1.

### 2.2 Assessment of inflammation and nutritional indices

To comprehensively investigate the influence of inflammation and nutrition on health outcomes, this study incorporated seven indices related to inflammatory and nutritional status for comparative analysis. These included five composite indices and two conventional indices: ALI, CLR, NPAR, NLR, SII, CRP, and ALB. The formulae for calculating the composite indices are as follows:


$$ALI=\frac{\mathrm{Weight}\;\left(\mathrm{kg}\right)}{\mathrm{Height}\;{\left(m\right)}^{2}}\times \mathrm{Albumin}\;\left(g/\mathrm{dL}\right)\times \frac{\mathrm{Lymphocyte}\;\left(1000\;\mathrm{cells}/\mathrm{\mu L}\right)}{\mathrm{Neutrophil}\;\left(1000\;\mathrm{cells}/\mathrm{\mu L}\right)}$$

$$CLR=\frac{CRP\;\left(\mathrm{mg}/\mathrm{dL}\right)}{\mathrm{Lymphocyte}\;\left(1000\;\mathrm{cells}/\mathrm{\mu L}\right)}$$

$$\mathrm{NPAR}=\frac{\mathrm{Neutrophil}\;\left(1000\;\mathrm{cells}/\mathrm{\mu L}\right)}{\mathrm{Albumin}\;\left(g/\mathrm{dL}\right)}$$

$$NLR=\frac{\mathrm{Neutrophil}\;\left(1000\;\mathrm{cells}/\mathrm{\mu L}\right)}{\mathrm{Lymphocyte}\;\left(1000\;\mathrm{cells}/\mathrm{\mu L}\right)}$$

$$SII=\frac{\mathrm{Neutrophil}\;\left(1000\;\mathrm{cells}/\mathrm{\mu L}\right)\times \mathrm{Platelet}\;\left(1000\;\mathrm{cells}/\mathrm{\mu L}\right)}{\mathrm{Lymphocyte}\;\left(1000\;\mathrm{cells}/\mathrm{\mu L}\right)}$$

Subsequently, the most effective index (highest AUC value) was selected using time-dependent receiver operating characteristic (ROC) analysis. Based on the quartiles of this indicator, participants were divided into four groups (Q1, Q2, Q3, Q4), with the first quartile group (Q1) serving as the reference group.

### 2.3 Assessment of mortality rates

Mortality data was linked to the NHANES dataset using identifiers provided by NCHS. This linkage utilized the National Death Index (NDI) to ascertain participants’ survival status, with follow-up data complete through December 31, 2019. Participants not found in the NDI were considered to be alive. Mortality assessment was conducted using the latest available NDI public mortality dataset, with causes of death classified according to the International Classification of Diseases, Tenth Revision (ICD-10). Cardio-cerebrovascular disease-related death codes included I00-I09, I11, I13, I20-I51, I60-I69 (29).

### 2.4 Covariates

This study considered multiple variables that could influence the relationship between inflammation, nutritional status, and the risk of cardio-cerebrovascular and all-cause mortality. These variables encompassed a range of demographic characteristics such as age, gender, race, marital status, education level, and poverty-income ratio, as well as smoking and drinking habits, and comorbid conditions like kidney disease, diabetes, hypertension, and cancer. Smoking history was determined by whether an individual had smoked more than 100 cigarettes in their lifetime, while drinking history was based on whether they had consumed at least one 12-ounce beer, a 5-ounce glass of wine, or a 1.5-ounce shot of liquor within a year. Cancer and cardio-cerebrovascular disease statuses were ascertained through self-reported questionnaires, kidney disease was identified by an eGFR <60 mL/min/1.73 m^2^ and a UACR >30 mg/g, and diabetes was diagnosed based on self-reported surveys, glycosylated hemoglobin levels, and fasting glucose levels. Further details are available on the NHANES official website.

### 2.5 Statistical analysis

The statistical analysis strictly followed the recommended design methods of the NHANES database, employing appropriate weights for each study component. For variables that conformed to a normal distribution, data were described as mean ± standard deviation ( $\bar{x}$ ± s); for non-normally distributed variables, frequency and percentage, median, and quartile ranges [M(P25, P75)] were used. Baseline differences in continuous variables were assessed using analysis of variance, while categorical variables were evaluated through the *χ*^2^ test, with results presented as percentages.

To determine the relationship between inflammation and nutritional statuses and mortality among MACCE survivors and to identify the most suitable indicators, the study initially employed Kaplan–Meier analysis to explore the relationships between seven inflammation and nutrition indices and all-cause and cardio-cerebrovascular mortality among MACCE survivors. ROC curve analysis was then used to compare the predictive value and efficacy of these indices for mortality. For the most effective index, a multivariable-adjusted Cox regression model further evaluated its association with all-cause and cardiovascular mortality among MACCE survivors, with results expressed as hazard ratios (HR) and 95% confidence intervals (CI). Three models were used for analysis: a crude model without any adjustments for confounders; Model 1 adjusted for demographic factors such as age, gender, race, poverty-income ratio (PIR), and education level; Model 2, which further adjusted for smoking, alcohol consumption, diabetes, hypertension, chronic kidney disease, and cancer history to control for these factors’ impact on the outcomes.

Additionally, the study combined restricted cubic spline (RCS) analysis and a multivariable-adjusted Cox regression model to evaluate the nonlinear relationships between inflammation, nutritional status and mortality rates among MACCE survivors, and determined the optimal number of nodes with Akaike information criterion. Recursive algorithms were used to determine inflection points in the presence of nonlinear relationships, further examining threshold effects. Subgroup analyses and sensitivity analyses were performed to evaluate factors that might affect model stability, ensuring the robustness and reliability of the findings. All analyses were conducted using R software (version 4.3.1), with a significance level set at *p* < 0.05 on a two-sided test.

## 3 Results

### 3.1 Baseline characteristics of participants

The study ultimately included 2,045 participants with an average age of 63.957 years, 54.657% of whom were male, and an average ALI of 63.396. Based on the quartiles of ALI, participants were subdivided into four groups (Q1, Q2, Q3, Q4). Compared to those in the lowest quartile (Q1 group), participants with higher ALI (Q2, Q3, and Q4 groups) were generally younger, had a higher proportion of females, and had higher average lymphocyte counts (LYM), body mass index (BMI), and albumin levels. However, they had a lower proportion of Caucasians, lower counts of neutrophils (NEU), C-reactive protein (CRP), CLR, NPAR, NLR, SII, and lower prevalence of chronic kidney disease and cancer, indicating lower risks of cardiovascular and all-cause mortality. There were no statistically significant differences among the four groups in terms of poverty-income ratio (PIR), educational level, platelet counts (PLT), smoking and drinking habits, or history of hypertension, diabetes, myocardial infarction, heart failure, and stroke. The detailed results are presented in Table 1.

**Table 1:** Characteristics of participants.

Characteristics	Quartiles of Ali total	Q1	Q2	Q3	Q4	*p*-value
		<37.7	> = 37.7, <54.1	> = 54.1, <75.8	> = 75.8	
Participants, *n*	2045	512	511	511	511	
ALI	63.396 ± 0.968	27.500 ± 0.389	46.118 ± 0.233	63.771 ± 0.361	109.981 ± 2.458	< 0.0001
Age, year	63.957 ± 0.473	69.243 ± 0.843	65.400 ± 0.682	62.310 ± 0.912	59.606 ± 0.750	< 0.0001
PIR	2.537 ± 0.056	2.503 ± 0.092	2.371 ± 0.093	2.642 ± 0.090	2.622 ± 0.105	0.088
Sex, *n* (%)						0.025
Male	1,219 (54.657)	333 (58.496)	329 (58.976)	287 (52.421)	270 (49.478)	
Female	826 (45.343)	179 (41.504)	182 (41.024)	224 (47.579)	241 (50.522)	
Race, *n* (%)						< 0.0001
Mexican American	252 (3.490)	42 (2.340)	64 (3.320)	82 (4.879)	64 (3.336)	
Non-Hispanic Black	390 (10.617)	55 (5.369)	73 (8.408)	93 (9.462)	169 (18.277)	
Non-Hispanic White	1,264 (79.294)	390 (87.090)	340 (82.782)	301 (78.704)	233 (69.896)	
Other Hispanic	82 (2.603)	13 (1.762)	20 (2.079)	25 (3.401)	24 (3.060)	
Other race	57 (3.996)	12 (3.438)	14 (3.411)	10 (3.553)	21 (5.432)	
Education, *n* (%)						0.068
< High school	409 (12.616)	95 (13.679)	117 (15.249)	100 (10.916)	97 (10.885)	
High school	893 (46.319)	232 (49.867)	226 (47.571)	226 (44.699)	209 (43.623)	
> High school	743 (41.065)	185 (36.455)	168 (37.180)	185 (44.385)	205 (45.492)	
LYM (1,000 cells/μL)	2.074 ± 0.031	1.419 ± 0.029	1.849 ± 0.028	2.171 ± 0.036	2.755 ± 0.103	< 0.0001
NEU (1,000 cells/μL)	4.527 ± 0.039	5.702 ± 0.099	4.751 ± 0.077	4.357 ± 0.076	3.464 ± 0.067	< 0.0001
PLT (1,000 cells/μL)	248.943 ± 2.133	245.292 ± 4.340	249.448 ± 4.149	253.349 ± 3.580	247.491 ± 5.032	0.493
Albumin, g/dL	4.177 ± 0.010	4.104 ± 0.020	4.169 ± 0.016	4.178 ± 0.019	4.247 ± 0.020	< 0.0001
CRP, mg/dL	0.587 ± 0.022	0.766 ± 0.062	0.496 ± 0.046	0.579 ± 0.048	0.521 ± 0.041	0.004
BMI, kg/m^2^	29.948 ± 0.197	26.690 ± 0.302	28.874 ± 0.332	31.011 ± 0.363	32.755 ± 0.375	< 0.0001
NPAR	1.093 ± 0.010	1.400 ± 0.025	1.147 ± 0.020	1.050 ± 0.018	0.820 ± 0.015	< 0.0001
CLR	0.339 ± 0.016	0.617 ± 0.050	0.277 ± 0.026	0.276 ± 0.023	0.212 ± 0.018	< 0.0001
NLR	2.558 ± 0.045	4.432 ± 0.119	2.620 ± 0.029	2.038 ± 0.025	1.367 ± 0.022	< 0.0001
SII	633.681 ± 13.271	1079.537 ± 39.627	650.913 ± 15.064	520.797 ± 10.605	337.988 ± 9.020	< 0.0001
Smoke, *n* (%)						0.436
No	744 (36.582)	169 (33.509)	178 (35.143)	194 (38.391)	203 (38.860)	
Yes	1,301 (63.418)	343 (66.491)	333 (64.857)	317 (61.609)	308 (61.140)	
Drinking, *n* (%)						0.395
No	300 (13.911)	64 (12.909)	76 (13.595)	80 (12.561)	80 (16.341)	
Yes	1745 (86.089)	448 (87.091)	435 (86.405)	431 (87.439)	431 (83.659)	
Hypertension, *n* (%)						0.632
No	470 (27.187)	121 (25.569)	126 (27.049)	119 (30.111)	104 (25.961)	
Yes	1,575 (72.813)	391 (74.431)	385 (72.951)	392 (69.889)	407 (74.039)	
DM, *n* (%)						0.168
No	1,297 (67.804)	354 (72.027)	328 (66.770)	298 (63.959)	317 (68.716)	
Yes	748 (32.196)	158 (27.973)	183 (33.230)	213 (36.041)	194 (31.284)	
CKD, *n* (%)						< 0.0001
No	1,074 (59.189)	210 (47.208)	250 (54.173)	280 (63.275)	334 (70.318)	
Yes	971 (40.811)	302 (52.792)	261 (45.827)	231 (36.725)	177 (29.682)	
Cancer, *n* (%)						< 0.001
No	1,617 (79.238)	361 (70.924)	404 (79.870)	415 (81.062)	437 (84.144)	
Yes	428 (20.762)	151 (29.076)	107 (20.130)	96 (18.938)	74 (15.856)	
Myocardial infarction, *n* (%)						0.209
No	972 (46.143)	243 (47.308)	216 (42.554)	256 (49.991)	257 (45.510)	
Yes	1,064 (53.462)	266 (52.692)	294 (57.446)	252 (50.009)	252 (54.490)	
Heart failure, *n* (%)						0.177
No	1,304 (65.156)	298 (60.997)	338 (67.293)	331 (66.105)	337 (68.397)	
Yes	715 (33.822)	206 (39.003)	165 (32.707)	174 (33.895)	170 (31.603)	
Stroke, *n* (%)						0.907
No	1,196 (59.632)	304 (59.794)	310 (60.656)	290 (58.178)	292 (60.019)	
Yes	846 (40.326)	208 (40.206)	200 (39.344)	220 (41.822)	218 (39.981)	
ALL-cause death, *n* (%)						< 0.0001
No	791 (44.281)	112 (27.175)	174 (38.282)	231 (51.298)	274 (57.986)	
Yes	1,254 (55.719)	400 (72.825)	337 (61.718)	280 (48.702)	237 (42.014)	
CVD-cause death, *n* (%)						< 0.001
No	1,528 (77.686)	346 (70.819)	367 (75.350)	391 (78.686)	424 (84.836)	
Yes	517 (22.314)	166 (29.181)	144 (24.650)	120 (21.314)	87 (15.164)	

The data are presented as the $\bar{x\;}$ ± s or *n* (%). Continuous variables were analysis of variance, and categorical variables were analysis of *χ*2 tests.

ALI, Advanced lung cancer inflammation index; PIR, Poverty-income ratio; LYM, lymphocyte; NEU, neutrophil; PLT, platelet; CRP, C-Reactive Protein; BMI, Body mass index; NPAR, Neutrophil to Serum Albumin Ratio; CLR, C-reactive protein to Lymphocyte Ratio; NLR, Neutrophil to Lymphocyte Ratio; SII, Systemic Immune-Inflammation Index; DM, Diabetes Mellitus; CKD, Chronic Kidney Disease; CVD, Cardiovascular Disease.

### 3.2 Diagnostic efficacy of indicators

**Figure 2:**
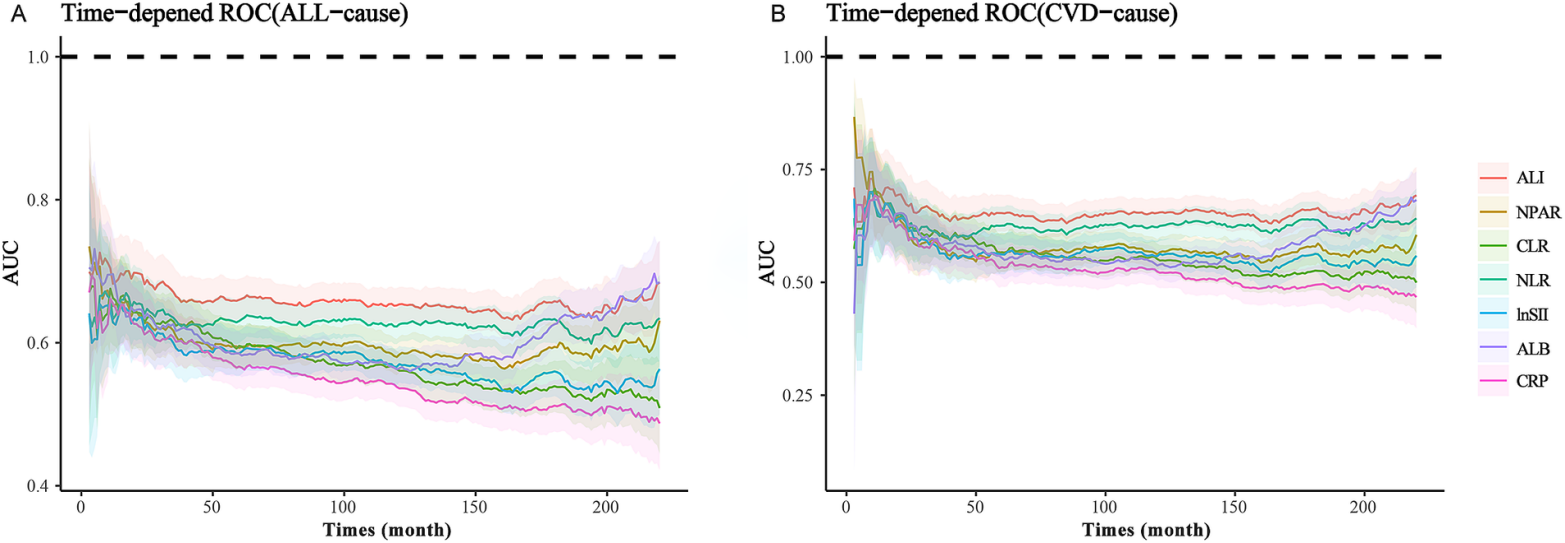
ROC analysis of various nutritional-inflammation indices: **(A)** ROC Curve of Nutritional-Inflammation Indices and ALL-cause mortality. **(B)** ROC Curve of Nutritional-Inflammation Indices and CVD-cause mortality.

To ascertain which inflammation and nutritional index most sensitively and effectively predicts mortality risk among MACCE survivors, we performed a time-dependent ROC analysis. The results revealed that the AUC values for ALI were superior in predicting both all-cause and cardiovascular mortality compared to composite indices such as NLR, NPAR, and CLR, as well as single indices like albumin and C-reactive protein. This proves that ALI is superior to the other indexes in predicting mortality risks. The specific results are illustrated in Figure 2.

### 3.3 Kaplan–Meier survival analysis

This study included data from 2,045 participants collected by NHANES from 1999 to 2010. During a median follow-up of 119 months for the participants at baseline, we identified 1,254 deaths from all causes, 517 deaths from CVD. To explore the health implications of inflammation, nutritional status on the MACCE population, the study initially employed Kaplan–Meier analysis to assess the association between seven inflammation, nutritional indices with all-cause and cardiovascular disease (CVD) mortality. The results revealed that the higher ALI level was significantly associated with lower risk of all-cause and CVD mortality (*P*_ALL_ < 0.0001, *P*_CVD_ < 0.0001). In contrast, higher levels of composite inflammatory indices such as CLR, NPAR, NLR, and SII were associated with a higher risk of mortality, specifically CLR (*P*_ALL_ = 0.001, *P*_CVD_ = 0.112); NPAR (*P*_ALL_ < 0.0001, *P*_CVD_ = 0.009); NLR (*P*_ALL_ < 0.0001, *P*_CVD_ < 0.0001); SII (*P*_ALL_ < 0.0001, *P*_CVD_ = 0.034). The individual markers CRP and ALB also showed correlations with mortality risks, with CRP (*P*_ALL_ = 0.002, *P*_CVD_ = 0.241) and ALB (*P*_ALL_ < 0.0001, *P*_CVD_ = 0.005) respectively. Detailed results are depicted in Supplementary Figure 1.

### 3.4 Relationship between ALI and mortality

Upon establishing ALI as the best predictor of long-term prognosis among MACCE survivors, we utilized a multivariable Cox regression model to quantify the relationship between ALI and both cardiovascular and all-cause mortality within the population. Model 2, incorporating a comprehensive set of covariates potentially affecting exposures and outcomes, served as the primary focus, showing that higher levels of ALI are associated with lower risks of all-cause and cardiovascular mortality among MACCE survivors. Specifically, for each unit increase in ALI, there is a 0.4% reduction in all-cause mortality and a 0.6% reduction in cardiovascular mortality risk, with results from Model 1 consistent with those of Model 2 (Table 2). As shown in Table 3, this trend was also significant across ALI quartiles; compared to the Q1 group, the hazard ratios (HR) for Q2, Q3, and Q4 were 0.730 (0.596, 0.894), 0.670 (0.561, 0.800), and 0.595 (0.467, 0.757) for all-cause mortality (*P* for trend <0.0001), and 0.717 (0.538, 0.957), 0.763 (0.571, 1.018), and 0.561 (0.376, 0.836) for cardiovascular mortality (*P* for trend = 0.006). Thus, compared to the Q1 group, the Q4 group’s all-cause and cardiovascular mortality rates decreased by 40.5 and 43.9%, respectively. Results of the Cox multivariable regression analysis for the other indices are available in Supplementary Tables S1, S2.

**Table 2:** Relationship between ALI and mortality in different models.

Outcome	Exposure	Crude model HR (95% CI)	*p*	Model 1 HR (95% CI)	*p*	Model 2 HR (95% CI)	*p*
ALL-cause	ALI	0.990 (0.987, 0.994)	<0.0001	0.996 (0.992, 0.999)	0.012	0.996 (0.993, 0.999)	0.022
CVD-cause		0.988 (0.982, 0.993)	<0.0001	0.994 (0.989, 0.999)	0.032	0.994 (0.990, 0.999)	0.041

Crude model: no-adjust; Model 1: age, sex, race, PIR, educational level; Model 2: age, sex, race, PIR, educational level, smoke, drinking, DM, Hypertension, CKD, cancer.

**Table 3:** Relationship between ALI quartile groups and mortality in different models.

Outcome	ALI groups	Crude model HR (95% CI)	*p*	Model 1 HR (95% CI)	*p*	Model 2 HR (95% CI)	*p*
ALL-cause	Q1	ref	ref	ref	ref	ref	ref
	Q2	0.653 (0.534, 0.797)	<0.0001	0.718 (0.590, 0.875)	<0.0001	0.730 (0.596, 0.894)	0.002
	Q3	0.466 (0.375, 0.578)	<0.0001	0.642 (0.542, 0.762)	<0.0001	0.670 (0.561, 0.800)	<0.0001
	Q4	0.367 (0.298, 0.453)	<0.0001	0.573 (0.450, 0.729)	<0.0001	0.595 (0.467, 0.757)	<0.0001
	*p* for trend		<0.0001		<0.0001		<0.0001
CVD-cause	Q1	ref	ref	ref	ref	ref	ref
	Q2	0.659 (0.497, 0.873)	0.004	0.728 (0.549, 0.965)	0.027	0.717 (0.538, 0.957)	0.024
	Q3	0.515 (0.383, 0.693)	<0.0001	0.741 (0.557, 0.986)	0.04	0.763 (0.571, 1.018)	0.066
	Q4	0.339 (0.233, 0.493)	<0.0001	0.559 (0.371, 0.842)	0.005	0.561 (0.376, 0.836)	0.004
	*p* for trend		<0.0001		0.005		0.006

Crude model: no-adjust; Model 1: age, sex, race, PIR, educational level; Model 2: age, sex, race, PIR, educational level, smoke, drinking, DM, Hypertension, CKD, cancer.

### 3.5 Nonlinear relationship between ALI and mortality

**Figure 3:**
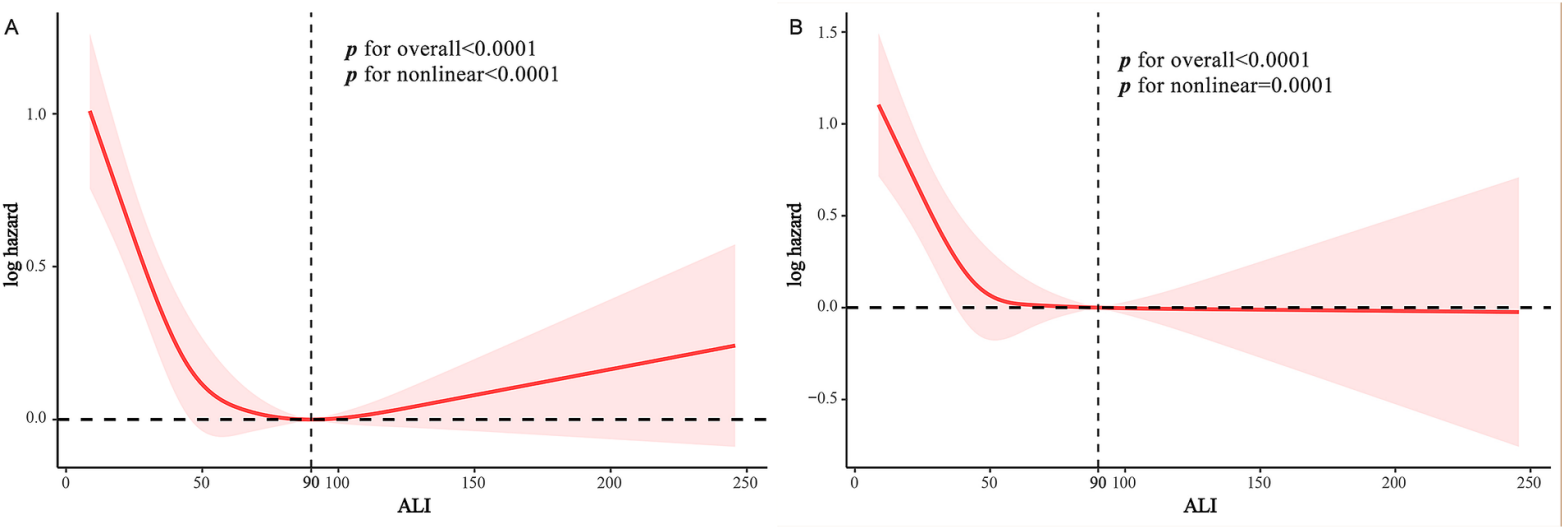
RCS curves of ALI impact on long-term ALL-cause and CVD-cause mortality in MACCE survivors: **(A)** RCS Curve of ALI and ALL-cause Mortality. **(B)** RCS curve of ALI and CVD-cause mortality.

Cox multivariable regression analysis indicated a nonlinear relationship between ALI and the long-term prognosis of MACCE survivors. To further elucidate this nonlinear association with survivorship, we employed RCS analysis to calculate inflection points while examining threshold effects. Our findings suggest a nonlinear association between ALI and both all-cause and cardiovascular mortality among MACCE survivors, as detailed in Figure 3. Recursive algorithms determined the inflection point for ALI with all-cause mortality at 90; to the left of this point, each unit increase in ALI resulted in a 1.3% decrease in all-cause and cardiovascular mortality risk among MACCE patients. However, beyond this inflection point, the relationship between ALI and both all-cause and cardiovascular mortality became nonsignificant (*p* > 0.05), as shown in Table 4.

**Table 4:** Threshold effect analysis of ALI on ALL-cause and CVD-cause mortality in MACCE survivors.

	Inflection point	HR (95% CI)	*p*	*p* for interaction
ALL-cause	<90	0.987 (0.983, 0.991)	<0.0001	< 0.0001
	> = 90	1.002 (0.999, 1.005)	0.141	
CVD-cause	<90	0.987 (0.980, 0.993)	<0.0001	0.012
	> = 90	1.002 (0.998, 1.006)	0.409	

### 3.6 Subgroup analysis

**Figure 4:**
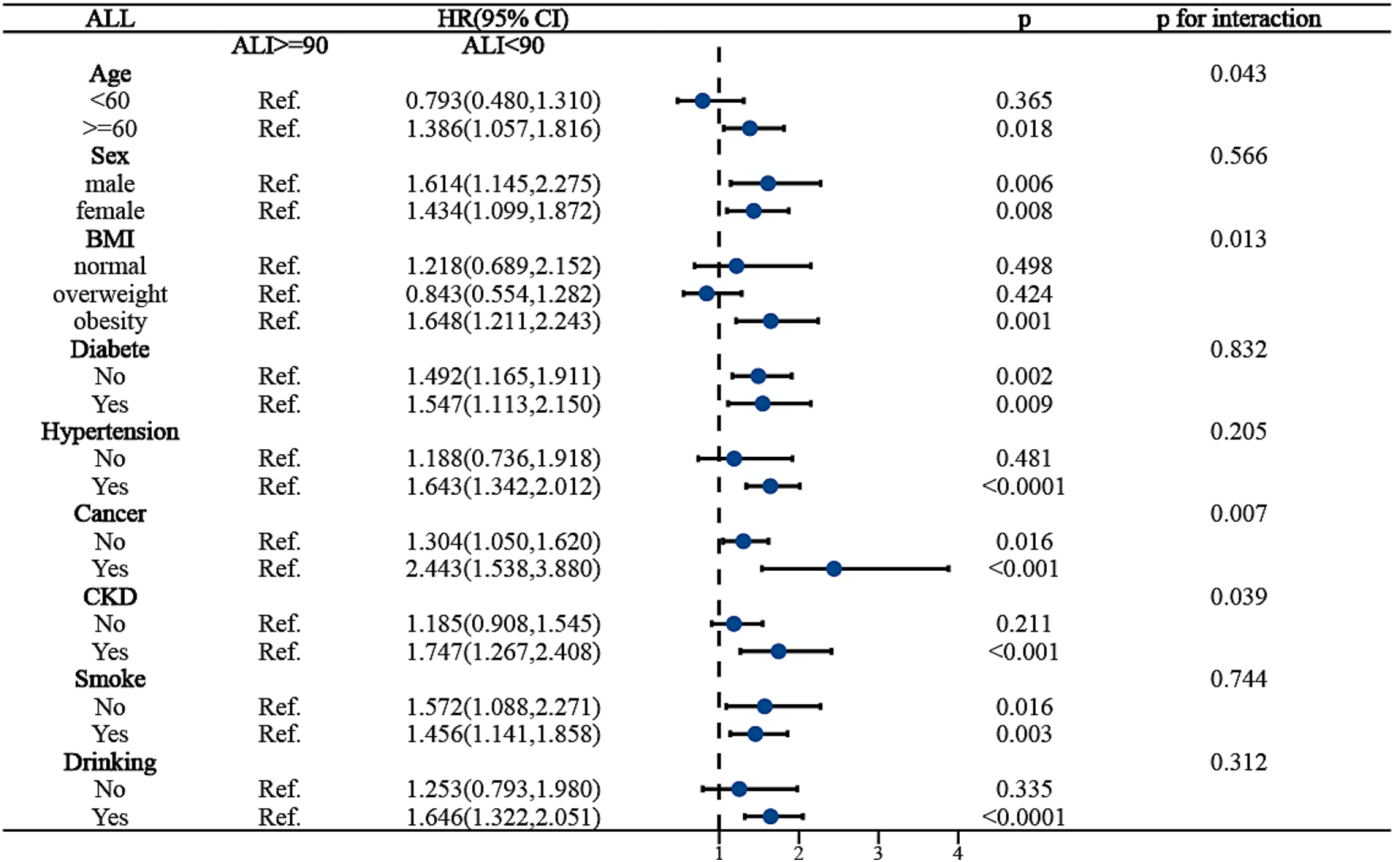
Subgroup analysis of ALI predicting ALL-cause mortality in populations with Non-fatal MACCE.

**Figure 5:**
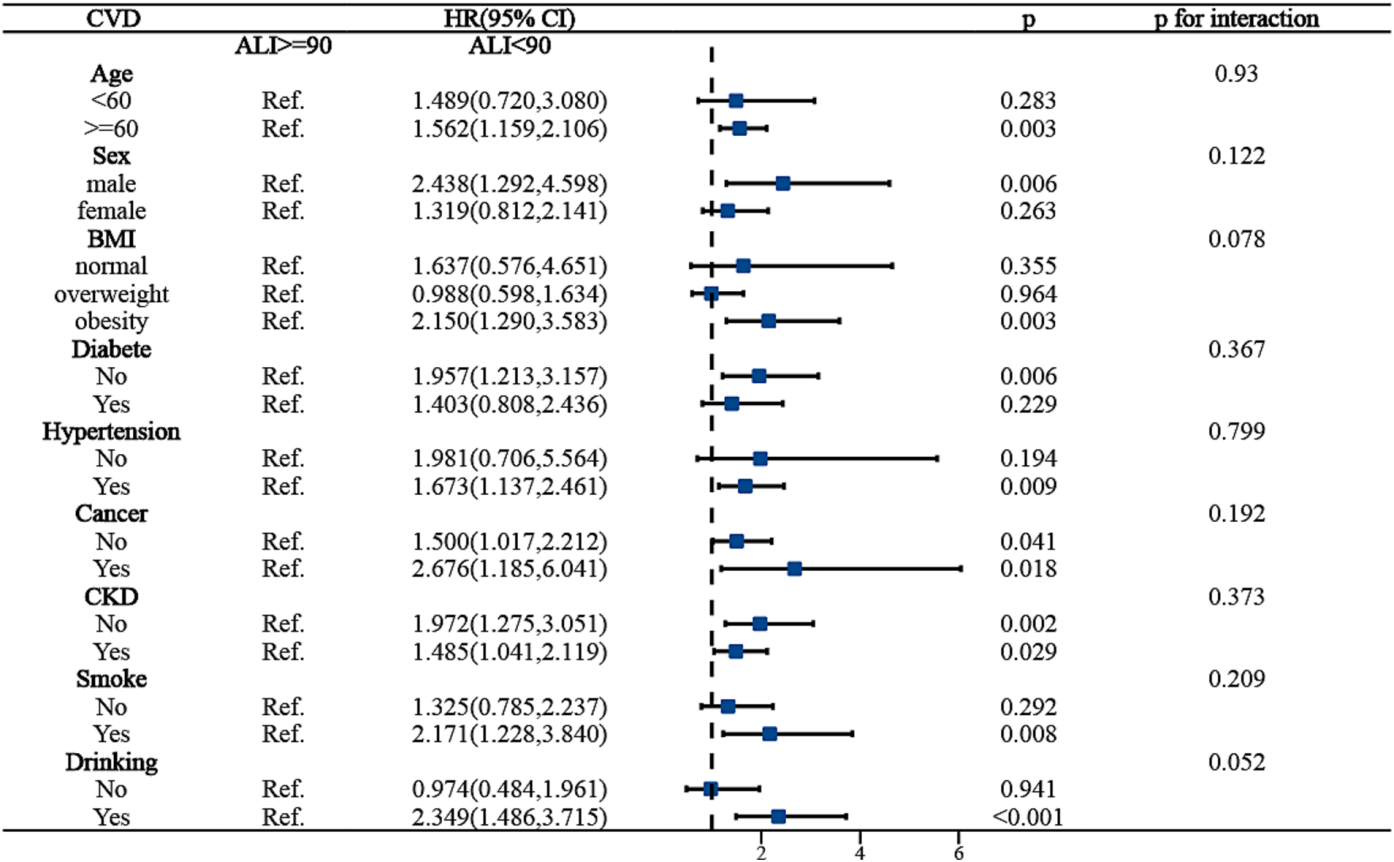
Subgroup analysis of ALI predicting CVD-cause mortality in populations with Non-fatal MACCE.

To ensure the stability of ALI’s predictive outcomes for the long-term prognosis of MACCE survivors, we conducted a subgroup analysis, basing it on the MACCE population to the right of the inflection point, and examining the death risk among different populations to the left of the inflection point. Figure 4 indicated that age, BMI, cancer, and chronic kidney disease might influence the predictive accuracy of ALI for all-cause mortality among MACCE survivors (*P* for interaction <0.05). Specifically, for individuals aged over 60, an ALI below 90 increased mortality risk by 38.6% [HR: 1.386, 95% CI (1.057, 1.186, *p* < 0.05)], but no such association was found in individuals under 60; for obese patients, compared to those with ALI above 90, all-cause mortality risk increased by 64.8% [HR: 1.648, 95% CI (1.211, 2.243, *p* < 0.05)], while no such relationship was observed in normal-weight or overweight individuals; patients with cancer and chronic kidney disease had 2.4 times [HR: 2.443, 95% CI (1.538, 3.880, *p* < 0.05)] and 74.7% [HR: 1.747, 95% CI (1.267, 2.408, *p* < 0.05)] higher all-cause mortality risk, respectively, compared to those without these conditions. In the subgroup analysis for cardiovascular mortality risk among MACCE survivors, ALI demonstrated robust stability, showing no significant interactions (*P* for interaction >0.05), as shown in Figure 5. Overall, ALI is a robust and reliable predictor of mortality risk among MACCE survivors.

### 3.7 Sensitivity analysis

Considering the effects of age, cancer, and obesity, we excluded young adults younger than 40 years of age, individuals with comorbid cancers, and individuals with BMI ≥30 kg/m^2^, ultimately including 863 participants for the study. Similar to the main study, we analyzed the association between ALI and risk of mortality using three multivariate regression models. The results confirmed the persistence of the association between ALI and the risk of all-cause and cardiovascular mortality in MACCE survivors, suggesting that the association was stable, as detailed in Table 5.

**Table 5:** Relationship between ALI and mortality in middle-aged and elderly MACCE survivors without cancer and obesity.

Outcome	ALI groups	Crude model HR (95% CI)	*p*	Model 1 HR (95% CI)	*p*	Model 2 HR (95% CI)	*p*
ALL-cause	ALI	0.991 (0.988, 0.994)	<0.0001	0.994 (0.991, 0.998)	0.0007	0.995 (0.991, 0.998)	0.002
	Q1	ref	ref	ref	ref	ref	ref
	Q2	0.572 (0.459, 0.714)	<0.0001	0.641 (0.512, 0.802)	0.0001	0.620 (0.494, 0.778)	<0.0001
	Q3	0.557 (0.446, 0.695)	<0.0001	0.595 (0.475, 0.745)	<0.0001	0.602 (0.478, 0.758)	<0.0001
	Q4	0.398 (0.314, 0.505)	<0.0001	0.511 (0.399, 0.654)	<0.0001	0.516 (0.402, 0.662)	<0.0001
	*p* for trend		<0.0001		<0.0001		<0.0001
CVD-cause	ALI	0.991 (0.986, 0.995)	0.0001	0.994 (0.989, 0.999)	0.018	0.994 (0.989, 1.000)	0.034
	Q1	ref	ref	ref	ref	ref	ref
	Q2	0.597 (0.428, 0.831)	0.002	0.654 (0.467, 0.915)	0.013	0.648 (0.461, 0.911)	0.012
	Q3	0.607 (0.437, 0.843)	0.003	0.630 (0.451, 0.880)	0.007	0.663 (0.471, 0.931)	0.018
	Q4	0.380 (0.264, 0.548)	<0.0001	0.494 (0.338, 0.721)	0.0003	0.513 (0.349, 0.752)	0.0006
	*p* for trend		<0.0001		0.0006		0.002

Crude model: no-adjust; Model 1: age, sex, race, PIR, educational level; Model 2: age, sex, race, PIR, educational level, smoke, drinking, DM, Hypertension, CKD, cancer.

## 4 Discussion

To our knowledge, this is the first study utilizing comprehensive and publicly accessible representative cohort data to compare the effects of ALI and alternative inflammation and nutritional indices (CLR, NPAR, NLR, SII, CRP, ALB) on all-cause and cardiovascular mortality in MACCE survivors. Our findings demonstrate a significant association between increased levels of ALI and ALB and reduced risks of all-cause and cardiovascular mortality. Conversely, elevated CLR, NPAR, NLR, SII, and CRP indices were correlated with higher mortality risks. Moreover, ROC results indicate that ALI is notably more effective in predicting risks of all-cause and cardiovascular mortality than other indices. RCS results establish a non-linear L-shaped relationship between ALI and all-cause mortality risk, with the lowest risk at an ALI of 90. To the left of this inflection point, each unit increase in ALI reduces all-cause and cardiovascular mortality risk by 1.3%. It should be noted that beyond this inflection point, the risk may increase by 0.2%, though this is not statistically significant. Subgroup analyses suggest potential interactions between age, BMI, cancer, and chronic kidney disease with ALI’s predictive accuracy for all-cause mortality; however, ALI’s robustness in predicting cardiovascular mortality risk shows no significant interactions.

Myocardial infarction, heart failure, and stroke are clinically significant cardio-cerebrovascular events. Globally, these events pose high mortality risks, and effective risk management for survivors is crucial for improving prognosis and enhancing quality of life. Recent insights recognize immune-inflammatory responses as a common critical pathological link among these events. Circulating immune cells such as macrophages, lymphocytes, and neutrophils and their secreted inflammatory mediators are extensively involved in the pathogenesis of atherosclerosis, endothelial dysfunction, and cardiac remodeling (7–10). Chronic inflammation can disrupt the intestinal microenvironment, leading to edema and permeability changes in the local gastrointestinal mucosa, affecting nutrient absorption.

Additionally, chronic inflammation can cause muscle wasting and insulin resistance, increases the risk of osteoporosis, accelerating the onset of malnutrition in survivors of cardio-cerebrovascular events, typically manifesting as weight and muscle mass loss and a frailty state, further increasing mortality risks (30–33). Notably, malnutrition can also influence inflammatory responses, creating a vicious cycle (34). Therefore, a comprehensive consideration of inflammation and nutritional status is vital for accurately managing mortality risks in survivors of cardio-cerebrovascular events.

ALI, calculated from NLR, ALB, and BMI, integrates both inflammatory and nutritional statuses. Our study preliminarily establishes that higher ALI is significantly associated with reduced all-cause and cardiovascular mortality rates, exhibiting a non-linear L-shaped relationship. This association likely reflects the organism’s immune-inflammatory and nutritional statuses. Neutrophils play a significant role in inflammation and innate immunity, while lymphocytes represent the adaptive immune system level, both closely related to the prognosis of cardio-cerebrovascular diseases (35). Their ratio, NLR, predicts mortality risk in patients with myocardial infarction, heart failure, and stroke (36–38). Serum ALB and related composite indicators are commonly used in clinical evaluation of nutritional status. Low levels of ALB not only indicates malnutrition, but also is related to inflammatory reaction, which can increase the risk of circulatory thromboembolism, and is a common biomarker for risk stratification of cardio-cerebrovascular diseases (39–41). Previous research has demonstrated that malnutrition states such as hypoalbuminemia and cardiac cachexia increase the risk of all-cause mortality among survivors of MACCE (42–44). In this study, the mean serum Neutrophil-to-Lymphocyte Ratio (NLR) of participants was 2.558, significantly higher than the average NLR of a healthy American population reported in previous studies, with an average serum Albumin (ALB) level of 4.177 g/dL. Our findings reveal a significant decrease in NLR levels from quartile 1 (Q1) to quartile 4 (Q4) among participants, accompanied by a notable increase in ALB levels, correlating with a substantial reduction in both all-cause and cardiovascular mortality. This suggests that reductions in NLR or increases in ALB levels are associated with decreased mortality risks among survivors of cardiovascular and cerebrovascular events.

BMI is a common indicator for assessing weight and nutritional status, typically defining obesity at a BMI ≥30 kg/m^2^ and underweight at BMI <18.5 kg/m^2^. Obesity is a risk factor for multiple cardiovascular and cerebrovascular diseases, such as myocardial infarction, heart failure, and stroke (45). However, multiple studies have indicated that, compared to those with healthy weight, individuals who are overweight or obese have a lower risk of mortality from these diseases, a phenomenon known as the “obesity paradox” (46, 47). Our study also finds that an increase in BMI is associated with reduced mortality risk. Subgroup analysis reveals that, compared to individuals with ALI greater than 90, obese patients had a 64.8% higher risk of all-cause mortality.

Further studies have pointed out a nonlinear relationship between BMI and mortality in patients with cardiovascular and cerebrovascular diseases. For instance, the relationship between BMI and all-cause mortality in heart failure patients exhibits a U-shape, with decreased mortality risk in overweight, Class I and II obesity compared to healthy weight; however, Class III obesity (BMI ≥40 kg/m^2^) shows a higher risk than that observed in the overweight category (48). An inverse J-shaped relationship was also found between BMI of 23.07 kg/m^2^ and the lowest risk of all-cause mortality in stroke patients (49). It’s noteworthy that high mortality risks associated with being underweight (BMI <18.5 kg/m^2^) in patients with cardiovascular and cerebrovascular diseases have been consistently observed in multiple studies (47–50). Based on this evidence, BMI may play a significant role in the L-shaped relationship between ABI and the all-cause and cardiovascular mortality among MACCE survivors. It’s important to note that BMI, as an indicator of weight status, has its limitations as it does not differentiate between fat mass, muscle mass, or edema-induced weight gain. Some researchers have proposed the use of a visceral obesity index (CVAI) to more accurately assess visceral fat content and identify potential risk for cardiovascular events (51). In addition, studies suggest that factors such as cardioprotective medications, duration of obesity, and smoking status may play roles in the obesity paradox (52).

In recent years, patients with cardiovascular diseases who also suffer from chronic kidney disease, metabolic disorders, and cancers have garnered increasing attention from scholars. The American Heart Association (AHA) has introduced the concept of the Cardiovascular-Kidney-Metabolic (CKM) syndrome, a cluster of interrelated diseases caused by the interactions between obesity, diabetes, chronic kidney disease, and cardiovascular conditions such as heart failure, coronary artery disease, and stroke, emphasizing the importance of managing cardiovascular diseases in conjunction with renal and metabolic disorders (53). Subgroup analyses in our study show that, compared to the MACCE survivors with ALI greater than 90, those with chronic kidney disease experienced a 74.7% increase in the risk of all-cause mortality. This may be related to the reduced renal function and excessive loss of albumin leading to malnutrition in patients with CKD. Additionally, subgroup analysis also indicates that MACCE survivors with concurrent cancer had a doubled risk of all-cause mortality, likely due to cancer being a chronic, debilitating illness often associated with varying degrees of frailty and malnutrition. Thus, it is imperative to enhance risk management for MACCE survivors with chronic kidney disease and cancer, with an emphasis on managing their comorbidities.

In conclusion, adequate nutritional support and anti-inflammatory treatments hold potential benefits for reducing the mortality risk associated with cardiovascular and cerebrovascular events. The Mediterranean diet pattern is a common anti-inflammatory diet, which consists of fats from fish, extra virgin olive oil, canola oil, and mixed nuts containing monounsaturated and polyunsaturated fatty acids (54). It has been shown by recent evidence to reduce the incidence of cardiovascular events and lower mortality risk (55–57). Other studies show that it is superior to a low-fat diet in preventing cardiovascular events (58). The anti-inflammatory diets play a role in modulating the intestinal microbiome, the intestinal mucosal barrier, and reducing endotoxin translocation into systemic circulation, thereby improving vascular endothelial function, ameliorating insulin resistance, and mitigating oxidative stress (59, 60). There is evidence of a bidirectional causal relationship between some gut flora and stroke, and regulating gut flora may reduce the potential risk (61). Currently, the Dietary Inflammatory Index (DII) has been utilized to guide risk prevention and control in relevant patients (62). Additionally, the use of low doses of medications such as colchicine, among other anti-inflammatory drugs, has been proven effective in reducing mortality risks in patients with cardiovascular and cerebrovascular diseases (63, 64). This underscores the importance of managing inflammation in risk management for survivors of cardiovascular events.

## 5 Strengths and limitations

The advantage of this study lies in using various statistical methods to evaluate and compare the sensitivity and efficacy of various inflammatory and nutritional indices, enhancing the objectivity and accuracy of the results. By employing multivariable-adjusted Cox analysis, subgroup analyses, and interaction analyses, the study minimizes confounding factors, bolstering the credibility of the findings. Additionally, as a novel index, ALI provides more precise guidance for risk management of cardio-cerebrovascular events due to its simple clinical operation and calculation, showing great potential for widespread application. Nonetheless, our study shares the limitations common to observational research, as it does not establish a causal relationship between ALI and mortality rates among cardio-cerebrovascular event survivors. In addition, the global applicability of the results needs to be further investigated because of the population limitations in the NHANES database. Further high-quality clinical evidence is needed to elucidate this causality. Future studies could explore changes in ALI over time and the predictive capability of dynamic ALI levels on mortality risks among survivors, aiming to better guide long-term risk management for patients.

## 6 Conclusion

The results of this study demonstrate that elevated ALI is significantly associated with reduced risks of all-cause and cardiovascular mortality among MACCE survivors. In a nationally representative sample of American MACCE survivors, we observed an L-shaped nonlinear relationship between ALI and both all-cause and cardiovascular mortality rates, with an inflection point (lowest mortality risk) at ALI 90. It should be noted that because of the limitations of this study, further investigation is still needed for the causal relationship between ALI and mortality risk and its applicability globally. Routine and dynamic monitoring of ALI is helpful for clinicians to assess the mortality risk in this population. Anti-inflammatory therapies and appropriate nutritional support are crucial for reducing mortality in these individuals.

## Data Availability

The datasets presented in this study can be found in online repositories. The names of the repository/repositories and accession number(s) can be found in the article/Supplementary material.

## References

[1] GBD 2019 Diseases and Injuries Collaborators. Global burden of 369 diseases and injuries in 204 countries and territories, 1990-2019: a systematic analysis for the global burden of disease study 2019. Lancet. (2020) 396:1204–22. doi: 10.1016/S0140-6736(20)30925-9 33069326 PMC7567026

[2] Mensah GA Fuster V Roth GA. A heart-healthy and stroke-free world: using data to inform global action. J Am Coll Cardiol. (2023) 82:2343–9. doi: 10.1016/j.jacc.2023.11.003, PMID: 38092508

[3] Mensah GA Fuster V Murray CJL Roth GA. Global burden of cardiovascular diseases and risks collaborators. Global burden of cardiovascular diseases and risks, 1990-2022. J Am Coll Cardiol. (2023) 82:2350–473. doi: 10.1016/j.jacc.2023.11.007, PMID: 38092509 PMC7615984

[4] Roger VL. Epidemiology of heart failure: a contemporary perspective. Circ Res. (2021) 128:1421–34. doi: 10.1161/CIRCRESAHA.121.318172 33983838

[5] Zhang H Jiao L Yang S Li H Jiang X Feng J. Brain-computer interfaces: the innovative key to unlocking neurological conditions. Int J Surg. (2024) 110:5745–62. doi: 10.1097/JS9.0000000000002022, PMID: 39166947 PMC11392146

[6] Tyrovola D Soulaidopoulos S Tsioufis C Lazaros G. The role of nutrition in cardiovascular disease: current concepts and trends. Nutrients. (2023) 15:1064. doi: 10.3390/nu15051064, PMID: 36904064 PMC10005442

[7] Koupenova M Clancy L Corkrey HA Freedman JE. Circulating platelets as mediators of immunity, inflammation, and thrombosis. Circ Res. (2018) 122:337–51. doi: 10.1161/CIRCRESAHA.117.310795, PMID: 29348254 PMC5777300

[8] Simats A Liesz A. Systemic inflammation after stroke: implications for post-stroke comorbidities. EMBO Mol Med. (2022) 14:e16269. doi: 10.15252/emmm.202216269, PMID: 35971650 PMC9449596

[9] Zuo W Sun R Ji Z Ma G. Macrophage-driven cardiac inflammation and healing: insights from homeostasis and myocardial infarction. Cell Mol Biol Lett. (2023) 28:81. doi: 10.1186/s11658-023-00491-4, PMID: 37858035 PMC10585879

[10] Carrillo-Salinas FJ Ngwenyama N Anastasiou M Kaur K Alcaide P. Heart inflammation: immune cell roles and roads to the heart. Am J Pathol. (2019) 189:1482–94. doi: 10.1016/j.ajpath.2019.04.009, PMID: 31108102 PMC6717912

[11] DeBerge M Chaudhary R Schroth S Thorp EB. Immunometabolism at the heart of cardiovascular disease. JACC Basic Transl Sci. (2023) 8:884–904. doi: 10.1016/j.jacbts.2022.12.010, PMID: 37547069 PMC10401297

[12] Raposeiras Roubín S Abu Assi E Cespón Fernandez M Barreiro Pardal C Lizancos Castro A Parada JA. Prevalence and prognostic significance of malnutrition in patients with acute coronary syndrome. J Am Coll Cardiol. (2020) 76:828–40. doi: 10.1016/j.jacc.2020.06.058, PMID: 32792081

[13] Wawrzeńczyk A Anaszewicz M Wawrzeńczyk A Budzyński J. Clinical significance of nutritional status in patients with chronic heart failure-a systematic review. Heart Fail Rev. (2019) 24:671–700. doi: 10.1007/s10741-019-09793-2, PMID: 31016426

[14] Zoellner ER Patterson MA Sharrief AZ Savitz SI Tucker WJ Miketinas DC. Dietary intake and quality among stroke survivors: NHANES 1999-2018. J Nutr. (2023) 153:3032–40. doi: 10.1016/j.tjnut.2023.08.015, PMID: 37598751

[15] He X Zhou T Yang Y Hong S Zhan J Hu Z. Advanced lung Cancer inflammation index, a new prognostic score, predicts outcome in patients with small-cell lung Cancer. Clin Lung Cancer. (2015) 16:e165–71. doi: 10.1016/j.cllc.2015.03.005, PMID: 25922292

[16] Song M Zhang Q Song C Liu T Zhang X Ruan G. The advanced lung cancer inflammation index is the optimal inflammatory biomarker of overall survival in patients with lung cancer. J Cachexia Sarcopenia Muscle. (2022) 13:2504–14. doi: 10.1002/jcsm.13032, PMID: 35833264 PMC9530543

[17] Wang X Wei C Fan W Sun L Zhang Y Sun Q. Advanced lung Cancer inflammation index for predicting prognostic risk for patients with acute coronary syndrome undergoing percutaneous coronary intervention. J Inflamm Res. (2023) 16:3631–41. doi: 10.2147/JIR.S421021, PMID: 37641701 PMC10460579

[18] Tu J Wu B Xiu J Deng J Lin S Lu J. Advanced lung cancer inflammation index is associated with long-term cardiovascular death in hypertensive patients: national health and nutrition examination study, 1999-2018. Front Physiol. (2023) 14:1074672. doi: 10.3389/fphys.2023.1074672, PMID: 37206362 PMC10189044

[19] Shi T Wang Y Peng Y Wang M Zhou Y Gu W. Advanced lung cancer inflammation index combined with geriatric nutritional risk index predict all-cause mortality in heart failure patients. BMC Cardiovasc Disord. (2023) 23:565. doi: 10.1186/s12872-023-03608-x, PMID: 37978441 PMC10655430

[20] Huang Y Wang X Li Z Yin X. A novel nutritional inflammation index for predicting mortality in acute ischemic stroke patients: insights into advanced lung cancer inflammation index from the medical information Mart for intensive care-IV database. Front Nutr. (2024) 11:1408372. doi: 10.3389/fnut.2024.1408372, PMID: 39036488 PMC11257925

[21] He L Xie H Du Y Xie X Zhang Y. The relationship between C-reactive protein to lymphocyte ratio and the prevalence of myocardial infarction in US adults: a cross-sectional study. Heliyon. (2023) 9:e17776. doi: 10.1016/j.heliyon.2023.e17776, PMID: 37483727 PMC10359823

[22] Kurkiewicz K Gąsior M Szyguła-Jurkiewicz BE. Markers of malnutrition, inflammation, and tissue remodeling are associated with 1-year outcomes in patients with advanced heart failure. Pol Arch Intern Med. (2023) 133:16411. doi: 10.20452/pamw.16411 36633195

[23] Feng KY Ambrosy AP Zhou Z Li D Kong J Zaroff JG. COAPT trial investigators. Association between serum albumin and outcomes in heart failure and secondary mitral regurgitation: the COAPT trial. Eur J Heart Fail. (2023) 25:553–61. doi: 10.1002/ejhf.2809, PMID: 36823954

[24] Tamaki S Nagai Y Shutta R Masuda D Yamashita S Seo M. Combination of neutrophil-to-lymphocyte and platelet-to-lymphocyte ratios as a novel predictor of cardiac death in patients with acute decompensated heart failure with preserved left ventricular ejection fraction: a multicenter study. J Am Heart Assoc. (2023) 12:e026326. doi: 10.1161/JAHA.122.026326, PMID: 36565197 PMC9973595

[25] Ma F Li L Xu L Wu J Zhang A Liao JMAF. The relationship between systemic inflammation index, systemic immune-inflammatory index, and inflammatory prognostic index and 90-day outcomes in acute ischemic stroke patients treated with intravenous thrombolysis. J Neuroinflammation. (2023) 20:220. doi: 10.1186/s12974-023-02890-y, PMID: 37777768 PMC10543872

[26] Cai X Song S Hu J Wang L Shen D Zhu Q. Systemic inflammation response index as a predictor of stroke risk in elderly patients with hypertension: a cohort study. J Inflamm Res. (2023) 16:4821–32. doi: 10.2147/JIR.S433190, PMID: 37901383 PMC10612501

[27] McCabe JJ Walsh C Gorey S Harris K Hervella P Iglesias-Rey R. C-reactive protein, Interleukin-6, and vascular recurrence after stroke: an individual participant data Meta-analysis. Stroke. (2023) 54:1289–99. doi: 10.1161/STROKEAHA.122.040529, PMID: 37026458

[28] Wang J Zhao J Ma Y Huang B Yuan D Han M. Frailty as a predictor of major adverse cardiac and cerebrovascular events after endovascular aortic aneurysm repair. J Vasc Surg. (2021) 74:442–450.e4. doi: 10.1016/j.jvs.2021.01.025, PMID: 33548426

[29] CDC. (2020). NCHS Data linked to NDI mortality files. Available at: https://www.cdc.gov/nchs/data-linkage/mortalityhtm (Accessed August 11, 2024).

[30] Yuxiu Y Ma X Gao F Liu T Deng J Wang Z. Combined effect of inflammation and malnutrition for long-term prognosis in patients with acute coronary syndrome undergoing percutaneous coronary intervention: a cohort study. BMC Cardiovasc Disord. (2024) 24:306. doi: 10.1186/s12872-024-03951-7, PMID: 38886675 PMC11181542

[31] Ma H Cai X Hu J Song S Zhu Q Zhang Y. Association of systemic inflammatory response index with bone mineral density, osteoporosis, and future fracture risk in elderly hypertensive patients. Postgrad Med. (2024) 136:406–16. doi: 10.1080/00325481.2024.2354158, PMID: 38753519

[32] Kumar S Conners KM Shearer JJ Joo J Turecamo S Sampson M. Frailty and metabolic vulnerability in heart failure: a community cohort study. J Am Heart Assoc. (2024) 13:e031616. doi: 10.1161/JAHA.123.031616, PMID: 38533960 PMC11262513

[33] Di Vincenzo O Pagano E Cervone M Natale R Morena A Esposito A. High nutritional risk is associated with poor functional status and prognostic biomarkers in stroke patients at admission to a rehabilitation unit. Nutrients. (2023) 15:4144. doi: 10.3390/nu15194144 37836427 PMC10574786

[34] Stumpf F Keller B Gressies C Schuetz P. Inflammation and nutrition: friend or foe? Nutrients. (2023) 15:1159. doi: 10.3390/nu15051159, PMID: 36904164 PMC10005147

[35] García-Escobar A Vera-Vera S Tébar-Márquez D Rivero-Santana B Jurado-Román A Jiménez-Valero S. Neutrophil-to-lymphocyte ratio an inflammatory biomarker, and prognostic marker in heart failure, cardiovascular disease and chronic inflammatory diseases: new insights for a potential predictor of anti-cytokine therapy responsiveness. Microvasc Res. (2023) 150:104598. doi: 10.1016/j.mvr.2023.104598, PMID: 37633337

[36] Li Q Yu Y Zhou YQ Zhao Y Wu J Wu YJ. Predictive value of neutrophil-to-lymphocyte ratio in coronary chronic total occlusion patients. J Geriatr Cardiol. (2024) 21:542–9. doi: 10.26599/1671-5411.2024.05.007, PMID: 38948892 PMC11211907

[37] Di Rosa M Sabbatinelli J Soraci L Corsonello A Bonfigli AR Cherubini A. Neutrophil-to-lymphocyte ratio (NLR) predicts mortality in hospitalized geriatric patients independent of the admission diagnosis: a multicenter prospective cohort study. J Transl Med. (2023) 21:835. doi: 10.1186/s12967-023-04717-z, PMID: 37990223 PMC10664513

[38] Chen S Cheng J Ye Q Ye Z Zhang Y Liu Y. Day 1 neutrophil-to-lymphocyte ratio (NLR) predicts stroke outcome after intravenous thrombolysis and mechanical thrombectomy. Front Neurol. (2022) 13:941251. doi: 10.3389/fneur.2022.941251, PMID: 36016545 PMC9396211

[39] Cai X Hu J Wen W Wang M Zhu Q Liu S. Association between the geriatric nutritional risk index and the risk of stroke in elderly patients with hypertension: a longitudinal and cohort study. Front Nutr. (2022) 9:1048206. doi: 10.3389/fnut.2022.1048206, PMID: 36562034 PMC9763600

[40] Sheinenzon A Shehadeh M Michelis R Shaoul E Ronen O. Serum albumin levels and inflammation. Int J Biol Macromol. (2021) 184:857–62. doi: 10.1016/j.ijbiomac.2021.06.140 34181998

[41] Ronit A Kirkegaard-Klitbo DM Dohlmann TL Lundgren J Sabin CA Phillips AN. Plasma albumin and incident cardiovascular disease: results from the CGPS and an updated Meta-analysis. Arterioscler Thromb Vasc Biol. (2020) 40:473–82. doi: 10.1161/ATVBAHA.119.313681 31852221

[42] Driggin E Cohen LP Gallagher D Karmally W Maddox T Hummel SL. Nutrition assessment and dietary interventions in heart failure: JACC review topic of the week. J Am Coll Cardiol. (2022) 79:1623–35. doi: 10.1016/j.jacc.2022.02.025, PMID: 35450580 PMC9388228

[43] Thuemmler RJ Pana TA Carter B Mahmood R Bettencourt-Silva JH Metcalf AK. Serum albumin and post-stroke outcomes: analysis of UK regional registry data, systematic review, and Meta-analysis. Nutrients. (2024) 16:1486. doi: 10.3390/nu16101486, PMID: 38794724 PMC11124370

[44] Bonilla-Palomas JL Gámez-López AL Castillo-Domínguez JC Moreno-Conde M López Ibáñez MC Alhambra Expósito R. Nutritional intervention in malnourished hospitalized patients with heart failure. Arch Med Res. (2016) 47:535–40. doi: 10.1016/j.arcmed.2016.11.005 28262195

[45] Kim MS Kim WJ Khera AV Kim JY Yon DK Lee SW. Association between adiposity and cardiovascular outcomes: an umbrella review and meta-analysis of observational and Mendelian randomization studies. Eur Heart J. (2021) 42:3388–403. doi: 10.1093/eurheartj/ehab454, PMID: 34333589 PMC8423481

[46] Mahajan R Stokes M Elliott A Munawar DA Khokhar KB Thiyagarajah A. Complex interaction of obesity, intentional weight loss and heart failure: a systematic review and meta-analysis. Heart. (2020) 106:58–68. doi: 10.1136/heartjnl-2019-314770, PMID: 31530572

[47] Akyea RK Ntaios G Doehner W. Obesity, metabolic health and clinical outcomes after incident cardiovascular disease: a nationwide population-based cohort study. J Cachexia Sarcopenia Muscle. (2023) 14:2653–62. doi: 10.1002/jcsm.13340, PMID: 37806948 PMC10751402

[48] Jones NR Ordóñez-Mena JM Roalfe AK Taylor KS Goyder CR Hobbs FR. Body mass index and survival in people with heart failure. Heart. (2023) 109:1542–9. doi: 10.1136/heartjnl-2023-322459, PMID: 37290898 PMC10579501

[49] Zan Y Xiong W Zhang X Han Y Cao C Hu H. Body mass index has a non-linear association with three-month outcomes in men with acute ischemic stroke: an analysis based on data from a prospective cohort study. Front Endocrinol. (2022) 13:1041379. doi: 10.3389/fendo.2022.1041379 PMC9792146 36578955

[50] Miwa K Nakai M Yoshimura S Sasahara Y Wada S Koge J. Clinical impact of body mass index on outcomes of ischemic and hemorrhagic strokes. Int J Stroke. (2024) 19:907–15. doi: 10.1177/17474930241249370, PMID: 38651751 PMC11408962

[51] Zhang H Zhan Q Dong F Gao X Zeng F Yao J. Associations of Chinese visceral adiposity index and new-onset stroke in middle-aged and older Chinese adults: an observational study. Lipids Health Dis. (2023) 22:74. doi: 10.1186/s12944-023-01843-x, PMID: 37337187 PMC10280837

[52] Simati S Kokkinos A Dalamaga M Argyrakopoulou G. Obesity paradox: fact or fiction? Curr Obes Rep. (2023) 12:75–85. doi: 10.1007/s13679-023-00497-1 36808566

[53] Ndumele CE Rangaswami J Chow SL Neeland IJ Tuttle KR Khan SS. Cardiovascular-kidney-metabolic health: a presidential advisory from the American Heart Association. Circulation. (2023) 148:1606–35. doi: 10.1161/CIR.0000000000001184, PMID: 37807924

[54] Martínez-González MÁ Hernández HA. Effect of the Mediterranean diet in cardiovascular prevention. Rev Esp Cardiol. (2024) 77:574–82. doi: 10.1016/j.rec.2024.01.006 38336153

[55] Billingsley HE Carbone S Lavie CJ. Dietary fats and chronic noncommunicable diseases. Nutrients. (2018) 10:1385. doi: 10.3390/nu10101385, PMID: 30274325 PMC6213917

[56] Billingsley HE Hummel SL Carbone S. The role of diet and nutrition in heart failure: a state-of-the-art narrative review. Prog Cardiovasc Dis. (2020) 63:538–51. doi: 10.1016/j.pcad.2020.08.004, PMID: 32798501 PMC7686142

[57] Karam G Agarwal A Sadeghirad B Jalink M Hitchcock CL Ge L. Comparison of seven popular structured dietary programmes and risk of mortality and major cardiovascular events in patients at increased cardiovascular risk: systematic review and network meta-analysis. BMJ. (2023) 380:e072003. doi: 10.1136/bmj-2022-072003, PMID: 36990505 PMC10053756

[58] Delgado-Lista J Alcala-Diaz JF Torres-Peña JD Quintana-Navarro GM Fuentes F Garcia-Rios A. Long-term secondary prevention of cardiovascular disease with a Mediterranean diet and a low-fat diet (CORDIOPREV): a randomised controlled trial. Lancet. (2022) 399:1876–85. doi: 10.1016/S0140-6736(22)00122-2 35525255

[59] Chen B Patel S Bao L Nadeem D Krittanawong C. Pro-inflammatory food, gut microbiota, and cardiovascular and pancreatic diseases. Biomol Ther. (2024) 14:210. doi: 10.3390/biom14020210, PMID: 38397447 PMC10886602

[60] Bagheri S Zolghadri S Stanek A. Beneficial effects of anti-inflammatory diet in modulating gut microbiota and controlling obesity. Nutrients. (2022) 14:3985. doi: 10.3390/nu14193985 36235638 PMC9572805

[61] Zhang H Jiang X Li A Wang X. Causal associations between gut microbiota and cerebrovascular diseases. World Neurosurg. (2024) 183:e587–97. doi: 10.1016/j.wneu.2023.12.150 38191059

[62] Marx W Veronese N Kelly JT Smith L Hockey M Collins S. The dietary inflammatory index and human health: an umbrella review of Meta-analyses of observational studies. Adv Nutr. (2021) 12:1681–90. doi: 10.1093/advances/nmab037, PMID: 33873204 PMC8483957

[63] Nelson K Fuster V Ridker PM. Low-dose colchicine for secondary prevention of coronary artery disease: JACC review topic of the week. J Am Coll Cardiol. (2023) 82:648–60. doi: 10.1016/j.jacc.2023.05.055, PMID: 37558377

[64] Goswami SK Ranjan P Dutta RK Verma SK. Management of inflammation in cardiovascular diseases. Pharmacol Res. (2021) 173:105912. doi: 10.1016/j.phrs.2021.105912, PMID: 34562603 PMC8541927

