# Supplementary figures and images for: Inflammation and nutritional status in relation to mortality risk from cardio-cerebrovascular events: evidence from NHANES
Source: Front Nutr. 2024 Dec 12;11:1504946. doi: 10.3389/fnut.2024.1504946 (PMC11669911; doi:10.3389/fnut.2024.1504946)

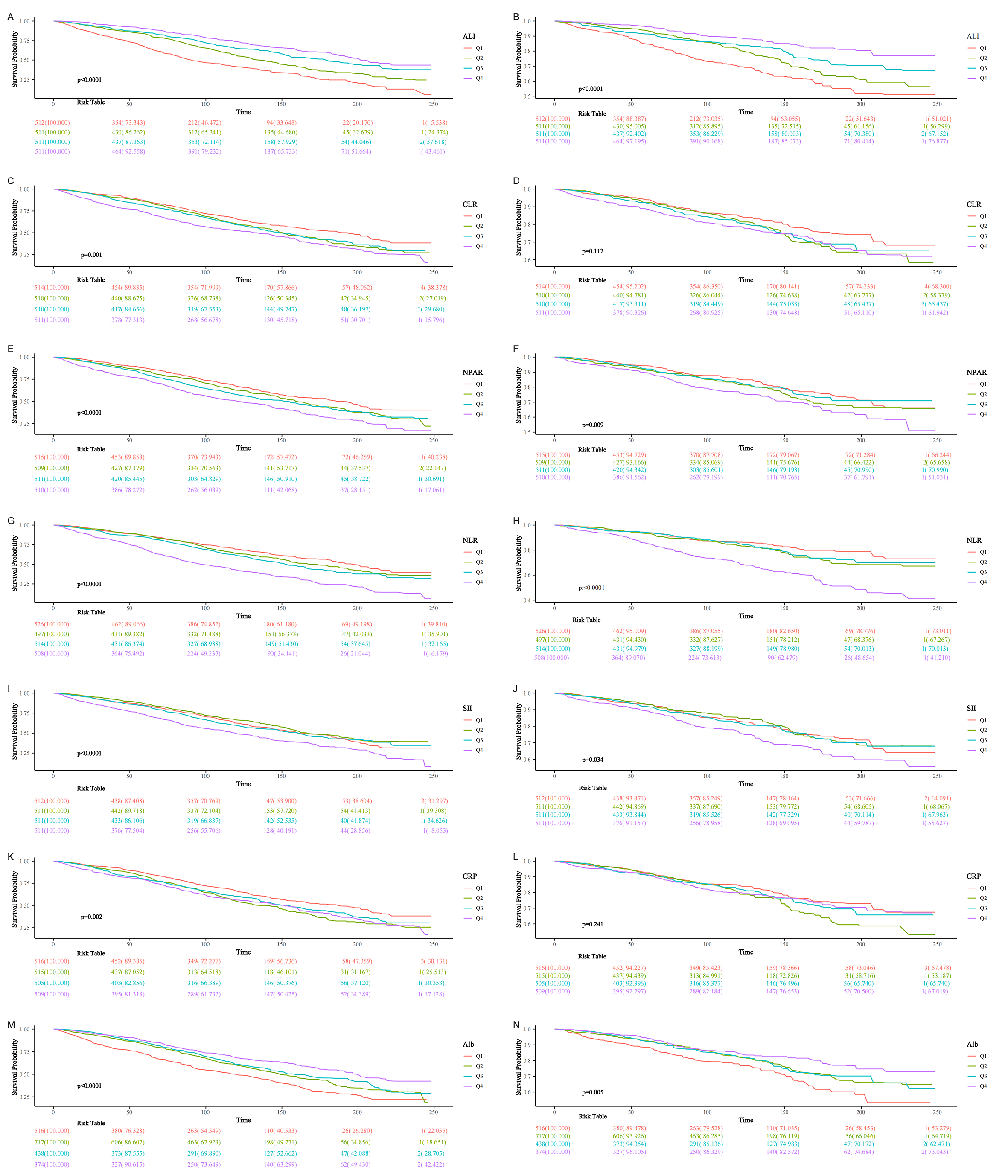

Supplement: Supplementary file 1 [file Image_1.tif]
