# Supplementary material for: Inflammation and nutritional status in relation to mortality risk from cardio-cerebrovascular events: evidence from NHANES
Source: Front Nutr. 2024 Dec 12;11:1504946. doi: 10.3389/fnut.2024.1504946 (PMC11669911; doi:10.3389/fnut.2024.1504946)
Supplement: Supplementary file 2 [file Table_1.docx]

Supplementary Material

**Supplementary Figure 1.** Kaplan-Meier survival curves of Nutrition-Inflammation Indices impact on long-term ALL-cause and CVD-cause mortality in MACCE survivors

**Supplementary Table S1.** Relationship between the seven inflammatory nutritional indices and ALL-cause mortality in different models.

**Supplementary Table S2.** Relationship between the seven inflammatory nutritional indices and CVD-cause mortality in different models.

**Supplementary Table S3.** Abbreviations.

**Supplementary Figure 1. Kaplan-Meier survival curves of Nutrition-Inflammation Indices impact on long-term ALL-cause and CVD-cause mortality in MACCE survivors ：**(A) Association between ALI and ALL-cause mortality in MACCE survivors. (B) Association between ALI and CVD-cause mortality in MACCE survivors. (C) Association between CLR and ALL-cause mortality in MACCE survivors. (D) Association between CLR and CVD-cause mortality in MACCE survivors. (E) Association between NPAR and ALL-cause mortality in MACCE survivors. (F) Association between NPAR and CVD-cause mortality in MACCE survivors. (G) Association between NLR and ALL-cause mortality in MACCE survivors. (H) Association between NLR and CVD-cause mortality in MACCE survivors. (I) Association between SII and ALL-cause mortality in MACCE survivors. (J) Association between SII and CVD-cause mortality in SII patients. (K) Association between CRP and ALL-cause mortality in MACCE survivors. (L) Association between CRP and CVD-cause mortality in MACCE survivors. (M) Association between ALB and ALL-cause mortality in MACCE survivors. (N) Association between ALB and CVD-cause mortality in MACCE survivors.

**Detailed images were shown in Supplementary Figure 1**

**Tabel S1. Relationship between the seven inflammatory nutritional indices and ALL-cause mortality in different models.**

| **Exposure** | **Crude Model**  **HR (95%CI)** | **P-value** | **Model 1**  **HR (95%CI)** | **P-value** | **Model 2**  **HR (95%CI)** | **P-value** |
| --- | --- | --- | --- | --- | --- | --- |
| **ALI** | 0.990(0.987,0.994) | <0.0001 | 0.996(0.992,0.999) | 0.012 | 0.996(0.993,0.999) | 0.022 |
| **ALI** |  |  |  |  |  |  |
| Q1 | ref | ref | ref | ref | ref | ref |
| Q2 | 0.653(0.534,0.797) | <0.0001 | 0.718(0.590,0.875) | <0.001 | 0.730(0.596,0.894) | 0.002 |
| Q3 | 0.466(0.375,0.578) | <0.0001 | 0.642(0.542,0.762) | <0.0001 | 0.670(0.561,0.800) | <0.0001 |
| Q4 | 0.367(0.298,0.453) | <0.0001 | 0.573(0.450,0.729) | <0.0001 | 0.595(0.467,0.757) | <0.0001 |
| **P for trend** |  | <0.0001 |  | <0.0001 |  | <0.0001 |
| **NPAR** | 1.636(1.364,1.962) | <0.0001 | 1.938(1.569,2.393) | <0.0001 | 1.799(1.449,2.233) | <0.0001 |
| **NPAR** |  |  |  |  |  |  |
| Q1 | ref | ref | ref | ref | ref | ref |
| Q2 | 1.208(0.972,1.503) | 0.089 | 0.984(0.820,1.180) | 0.862 | 0.980(0.819,1.173) | 0.825 |
| Q3 | 1.311(1.061,1.620) | 0.012 | 1.231(1.003,1.511) | 0.046 | 1.172(0.962,1.428) | 0.114 |
| Q4 | 1.757(1.445,2.136) | <0.0001 | 1.753(1.413,2.176) | <0.0001 | 1.633(1.315,2.029) | <0.0001 |
| **P for trend** |  | <0.0001 |  | <0.0001 |  | <0.0001 |
| **CLR** | 1.191(1.124,1.262) | <0.0001 | 1.150(1.087,1.216) | <0.0001 | 1.159(1.100,1.222) | <0.0001 |
| **CLR** |  |  |  |  |  |  |
| Q1 | ref | ref | ref | ref | ref | ref |
| Q2 | 1.277(1.028,1.586) | 0.027 | 1.110(0.909,1.355) | 0.305 | 1.156(0.943,1.418) | 0.162 |
| Q3 | 1.282(1.019,1.613) | 0.034 | 1.148(0.926,1.423) | 0.208 | 1.171(0.939,1.461) | 0.161 |
| Q4 | 1.607(1.279,2.018) | <0.0001 | 1.546(1.272,1.879) | <0.0001 | 1.481(1.211,1.810) | <0.001 |
| **P for trend** |  | <0.0001 |  | <0.0001 |  | <0.001 |
| **NLR** | 1.211(1.167,1.258) | <0.0001 | 1.168(1.117,1.221) | <0.0001 | 1.156(1.107,1.208) | <0.0001 |
| **NLR** |  |  |  |  |  |  |
| Q1 | ref | ref | ref | ref | ref | ref |
| Q2 | 1.169(0.925,1.477) | 0.192 | 1.102(0.912,1.332) | 0.313 | 1.100(0.912,1.327) | 0.317 |
| Q3 | 1.323(1.066,1.642) | 0.011 | 1.088(0.886,1.336) | 0.422 | 1.054(0.851,1.305) | 0.629 |
| Q4 | 2.318(1.900,2.828) | <0.0001 | 1.664(1.343,2.063) | <0.0001 | 1.601(1.286,1.994) | <0.0001 |
| **P for trend** |  | <0.0001 |  | <0.0001 |  | <0.0001 |
| **ALB** | 0.481(0.384,0.603) | <0.0001 | 0.486(0.375,0.631) | <0.0001 | 0.534(0.408,0.700) | <0.0001 |
| **ALB** |  |  |  |  |  |  |
| Q1 | ref | ref | ref | ref | ref | ref |
| Q2 | 0.718(0.603,0.855) | <0.001 | 0.659(0.560,0.776) | <0.0001 | 0.685(0.572,0.821) | <0.0001 |
| Q3 | 0.655(0.543,0.791) | <0.0001 | 0.658(0.540,0.801) | <0.0001 | 0.681(0.549,0.845) | <0.001 |
| Q4 | 0.499(0.398,0.625) | <0.0001 | 0.545(0.424,0.699) | <0.0001 | 0.584(0.454,0.750) | <0.0001 |
| **P for trend** |  | <0.0001 |  | <0.0001 |  | <0.0001 |
| **CRP** | 1.094(1.029,1.164) | 0.004 | 1.110(1.051,1.172) | <0.001 | 1.117(1.061,1.176) | <0.0001 |
| **CRP** |  |  |  |  |  |  |
| Q1 | ref | ref | ref | ref | ref | ref |
| Q2 | 1.426(1.169,1.739) | <0.001 | 1.256(1.035,1.524) | 0.021 | 1.284(1.052,1.567) | 0.014 |
| Q3 | 1.258(1.011,1.566) | 0.039 | 1.162(0.960,1.406) | 0.123 | 1.158(0.948,1.414) | 0.151 |
| Q4 | 1.398(1.125,1.736) | 0.002 | 1.570(1.282,1.922) | <0.0001 | 1.504(1.221,1.852) | <0.001 |
| **P for trend** |  | 0.011 |  | <0.0001 |  | <0.001 |
| **Ln(SII)** | 1.337(1.155,1.549) | <0.001 | 1.255(1.080,1.458) | 0.003 | 1.226(1.050,1.430) | 0.01 |
| **SII** |  |  |  |  |  |  |
| Q1 | ref | ref | ref | ref | ref | ref |
| Q2 | 0.882(0.695,1.119) | 0.301 | 0.952(0.762,1.189) | 0.664 | 0.948(0.759,1.184) | 0.639 |
| Q3 | 1.023(0.824,1.269) | 0.839 | 1.042(0.852,1.275) | 0.686 | 1.020(0.835,1.245) | 0.85 |
| Q4 | 1.494(1.230,1.816) | <0.0001 | 1.435(1.160,1.775) | <0.001 | 1.399(1.128,1.736) | 0.002 |
| **P for trend** |  | <0.0001 |  | <0.001 |  | <0.001 |

Note: Crude Mode l: No-adjust; Model 1: age, sex, race, PIR, educational level; Model 2: age, sex, race, PIR, educational level, smoke, drinking, DM, Hypertension, CKD, cancer.

**Tabel S2.** **Relationship between the seven inflammatory nutritional indices and CVD-cause mortality in different models.**

| **Exposure** | **Crude Model**  **HR (95%CI)** | **P-value** | **Model 1**  **HR (95%CI)** | **P-value** | **Model 2**  **HR (95%CI)** | **P-value** |
| --- | --- | --- | --- | --- | --- | --- |
| **ALI** | 0.988(0.982,0.993) | <0.0001 | 0.994(0.989,0.999) | 0.032 | 0.994(0.990,0.999) | 0.041 |
| **ALI** |  |  |  |  |  |  |
| Q1 | ref | ref | ref | ref | ref | ref |
| Q2 | 0.659(0.497,0.873) | 0.004 | 0.728(0.549,0.965) | 0.027 | 0.717(0.538,0.957) | 0.024 |
| Q3 | 0.515(0.383,0.693) | <0.0001 | 0.741(0.557,0.986) | 0.04 | 0.763(0.571,1.018) | 0.066 |
| Q4 | 0.339(0.233,0.493) | <0.0001 | 0.559(0.371,0.842) | 0.005 | 0.561(0.376,0.836) | 0.004 |
| **P for trend** |  | <0.0001 |  | 0.005 |  | 0.006 |
| **NPAR** | 1.523(1.194,1.944) | <0.001 | 1.814(1.341,2.453) | <0.001 | 1.674(1.239,2.260) | <0.001 |
| **NPAR** |  |  |  |  |  |  |
| Q1 | ref | ref | ref | ref | ref | ref |
| Q2 | 1.189(0.844,1.675) | 0.323 | 0.944(0.711,1.254) | 0.692 | 0.945(0.713,1.253) | 0.695 |
| Q3 | 1.064(0.772,1.468) | 0.703 | 0.979(0.715,1.342) | 0.897 | 0.924(0.674,1.267) | 0.622 |
| Q4 | 1.550(1.183,2.031) | 0.001 | 1.553(1.155,2.088) | 0.004 | 1.450(1.086,1.937) | 0.012 |
| **P for trend** |  | 0.006 |  | 0.006 |  | 0.023 |
| **CLR** | 1.190(1.102,1.285) | <0.0001 | 1.139(1.064,1.219) | <0.001 | 1.155(1.078,1.239) | <0.0001 |
| **CLR** |  |  |  |  |  |  |
| Q1 | ref | ref | ref | ref | ref | ref |
| Q2 | 1.283(0.952,1.731) | 0.102 | 1.106(0.811,1.506) | 0.525 | 1.192(0.870,1.634) | 0.275 |
| Q3 | 1.258(0.910,1.740) | 0.165 | 1.121(0.809,1.553) | 0.494 | 1.159(0.829,1.622) | 0.388 |
| Q4 | 1.418(1.070,1.880) | 0.015 | 1.371(1.016,1.849) | 0.039 | 1.369(1.019,1.837) | 0.037 |
| **P for trend** |  | 0.023 |  | 0.05 |  | 0.059 |
| **NLR** | 1.223(1.161,1.288) | <0.0001 | 1.174(1.091,1.264) | <0.0001 | 1.161(1.080,1.249) | <0.0001 |
| **NLR** |  |  |  |  |  |  |
| Q1 | ref | ref | ref | ref | ref | ref |
| Q2 | 1.311(0.967,1.777) | 0.081 | 1.205(0.906,1.602) | 0.199 | 1.204(0.894,1.621) | 0.221 |
| Q3 | 1.210(0.881,1.664) | 0.239 | 0.951(0.690,1.310) | 0.757 | 0.915(0.658,1.272) | 0.596 |
| Q4 | 2.604(1.917,3.538) | <0.0001 | 1.763(1.245,2.496) | 0.001 | 1.725(1.221,2.436) | 0.002 |
| **P for trend** |  | <0.0001 |  | 0.005 |  | 0.008 |
| **ALB** | 0.529(0.375,0.747) | <0.001 | 0.545(0.356,0.833) | 0.005 | 0.581(0.382,0.884) | 0.011 |
| **ALB** |  |  |  |  |  |  |
| Q1 | ref | ref | ref | ref | ref | ref |
| Q2 | 0.715(0.539,0.950) | 0.021 | 0.654(0.483,0.885) | 0.006 | 0.680(0.496,0.934) | 0.017 |
| Q3 | 0.709(0.508,0.991) | 0.044 | 0.716(0.504,1.017) | 0.062 | 0.721(0.503,1.033) | 0.074 |
| Q4 | 0.535(0.379,0.753) | <0.001 | 0.590(0.408,0.854) | 0.005 | 0.626(0.430,0.911) | 0.014 |
| **P for trend** |  | <0.001 |  | 0.015 |  | 0.028 |
| **CRP** | 1.092(0.990,1.204) | 0.08 | 1.107(1.026,1.195) | 0.009 | 1.120(1.034,1.213) | 0.005 |
| **CRP** |  |  |  |  |  |  |
| Q1 | ref | ref | ref | ref | ref | ref |
| Q2 | 1.355(1.023,1.795) | 0.034 | 1.184(0.891,1.574) | 0.244 | 1.256(0.939,1.680) | 0.124 |
| Q3 | 1.147(0.893,1.475) | 0.283 | 1.063(0.814,1.387) | 0.654 | 1.066(0.808,1.407) | 0.652 |
| Q4 | 1.154(0.838,1.588) | 0.38 | 1.324(0.940,1.863) | 0.108 | 1.336(0.959,1.862) | 0.087 |
| **P for trend** |  | 0.536 |  | 0.183 |  | 0.189 |
| **Ln(SII)** | 1.352(1.100,1.662) | 0.004 | 1.254(1.011,1.556) | 0.039 | 1.221(0.984,1.514) | 0.069 |
| **SII** |  |  |  |  |  |  |
| Q1 | ref | ref | ref | ref | ref | ref |
| Q2 | 0.947(0.679,1.323) | 0.751 | 0.991(0.724,1.358) | 0.957 | 1.016(0.745,1.384) | 0.921 |
| Q3 | 1.022(0.757,1.381) | 0.885 | 1.033(0.773,1.381) | 0.827 | 1.025(0.766,1.372) | 0.868 |
| Q4 | 1.447(1.060,1.973) | 0.02 | 1.351(0.975,1.873) | 0.07 | 1.341(0.974,1.847) | 0.072 |
| **P for trend** |  | 0.012 |  | 0.055 |  | 0.066 |

Note: Crude Mode l: No-adjust; Model 1: age, sex, race, PIR, educational level; Model 2: age, sex, race, PIR, educational level, smoke, drinking, DM, Hypertension, CKD, cancer.

**Supplementary Table S3. Abbreviations.**

| **Abbreviations** | **Explaination** |
| --- | --- |
| NHANES | National Health and Nutrition Examination Survey |
| MACCE | major adverse cardiovascular and cerebrovascular events |
| NCHS | National Center for Health Statistics |
| NDI | National Death Index |
| ALI | Advanced Lung Cancer Inflammation Index |
| ALB | Albumin |
| CLR | C-reactive protein to Lymphocyte Ratio |
| NPAR | Neutrophil to Serum Albumin Ratio |
| NLR | Neutrophil-to-Lymphocyte Ratio |
| SII | Systemic Immune-Inflammation Index |
| CRP | C-reactive Protein |
| DM | Diabetes Mellitus |
| CKD | Chronic Kidney Disease |
| CVD | Cardiovascular disease |
| PIR | Poverty-Income Ratio |
| BMI | Body Mass Index |
| ROC | Receiver Operating Characteristic |
| RCS | Restricted Cubic Spline |
| HR | Hazard Ratios |
| CI | Confidence Intervals |
